# Outcomes of Glaucoma Referrals in Adults Aged 18 to 40 Years

**DOI:** 10.1001/jamanetworkopen.2024.57843

**Published:** 2025-02-06

**Authors:** Tanner Frediani, Kristy Yoo, Austin Cho, Jennifer Louie, Kent Nguyen, Grace Richter, John Shan, Benjamin Y. Xu

**Affiliations:** 1 Keck School of Medicine, University of Southern California, Los Angeles; 2 Vision Essentials Regional Office, Southern California Permanente Medical Group, Los Angeles; 3 Roski Eye Institute, Department of Ophthalmology, Keck School of Medicine, University of Southern California, Los Angeles; 4 Los Angeles General Medical Center, Southern California Permanente Medical Group, Los Angeles; 5 Panorama City Medical Center, Southern California Permanente Medical Group, Los Angeles

## Abstract

**Importance:**

While early detection of glaucoma is vital to prevent irreversible vision loss, there are sparse data on the effectiveness of glaucoma referrals and methods to establish evidence-based referral guidelines in large, diverse populations.

**Objective:**

To assess the prevalence and risk factors for diagnosed glaucoma and loss to follow-up among adult patients aged 18 to 40 years after a new diagnosis of referable glaucoma (ie, with glaucoma or suspected glaucoma).

**Design, Setting, and Participants:**

This retrospective cohort study included patients aged 18 to 40 years presenting to Kaiser Permanente Southern California, a large managed health care system, for first-time eye examinations between January 1, 2013, and December 31, 2018. Data analysis occurred between September 2022 and August 2024.

**Main Outcomes and Measures:**

The primary outcome was glaucoma diagnosed within 2 years of the first eye examination. The secondary outcome was the loss to follow-up, defined as failure to receive a glaucoma evaluation with visual field or optical coherence tomography testing within 2 years.

**Results:**

The cohort included 292 453 patients aged 18 to 40 years who underwent first-time eye evaluations (mean [SD] age, 29.8 [6.4] years). Among 12 050 identified patients with referable glaucoma (52.3% female), 6827 (56.7%) completed glaucoma evaluations, of whom 563 (8.2%) were diagnosed with glaucoma (344 [61.1%] with open angle, 28 [5.0%] with angle closure, 84 [14.9%] with secondary glaucoma, and 107 [19.0%] with unspecified glaucoma). On multivariable analysis, male sex (odds ratio [OR], 1.55 [95% CI, 1.07-2.27]), higher intraocular pressure (IOP) (OR, 1.19 [95% CI, 1.15-1.23] per 1 mm Hg), and greater cup-disc ratio (CDR) (OR, 1.53 [95% CI, 1.34-1.75] per 0.1 unit) were associated with greater odds of glaucoma. Dichotomized age, IOP, and CDR models stratified 51 of 1613 patients (3.2%) into the low-risk group and 202 of 1477 patients (13.7%) into the high-risk group. Being younger than 32 years and having an IOP less than 18 mm Hg and a CDR less than 0.7 yielded a negative predictive value of 98.2% for a glaucoma diagnosis.

**Conclusions and Relevance:**

In this cohort study, the diagnostic yield of glaucoma referrals was low among adults aged 18 to 40 years with first-time eye examinations. A simple risk-stratification strategy could help identify individuals with low and high risks of developing glaucoma, and adoption of evidence-based risk stratification and referral guidelines by health care systems and clinicians could improve equity of glaucoma care and use of eye-care resources.

## Key Points

**Question:**

What are the outcomes of glaucoma referrals in adults aged 18 to 40 years, and how can referral efficiency be improved?

**Findings:**

In this cohort study of 292 453 adults aged 18 to 40 years, only 8% of those referred for glaucoma evaluation were diagnosed with glaucoma within 2 years. A simple 3-variable model based on age, intraocular pressure, and cup-disc ratio stratified patients with low risk (3%) and high risk (14%) of being diagnosed with glaucoma.

**Meaning:**

The findings of this study suggest that the diagnostic yield of glaucoma evaluations in adults aged 18 to 40 years is low but that standardized referral guidelines can help risk stratify patients to ensure equitable eye care and efficient resource allocation.

## Teaser

This cohort study assesses the outcomes of glaucoma referrals in adults aged 18 to 40 years and the efficiency of models to stratify patients at risk of developing glaucoma.

## Introduction

Glaucoma is a major public health issue and the leading cause of irreversible vision loss worldwide.^1^ The burden of glaucoma on patients, clinicians, and health care systems is expected to increase rapidly as its global prevalence rises to 112 million people by 2040.^1^ Individuals are identified as being at risk for glaucoma (or who have suspected glaucoma) if they exhibit risk factors, such as an enlarged cup-disc ratio (CDR) or elevated intraocular pressure (IOP), on routine eye examination.^2,3^ Due to the irreversible nature of glaucomatous vision loss, the American Academy of Ophthalmology and the World Glaucoma Association emphasize the importance of referring individuals with suspected glaucoma for in-office glaucoma evaluation and diagnostic testing by an ophthalmologist to establish the presence or absence of the disease.^4,5^ However, there is a paucity of clear guidelines about referral criteria and evidence supporting the effectiveness of current referral practice patterns.

Earlier studies investigating the outcomes of glaucoma referrals were mostly conducted prior to the popularization of research using data from electronic health records (EHRs) and are limited by small sample sizes and homogeneous study populations.^6,7,8^ Therefore, further study of large, diverse patient populations is needed to establish methods for risk stratification and guidelines for glaucoma referrals. The importance of standardized referral guidelines is highlighted by glaring disparities in glaucoma detection and outcomes.^9^ For example, Black and Hispanic Americans are less likely to be evaluated or identified as at risk for glaucoma but are more likely to require surgery or develop blindness from glaucoma.^10,11,12^ Improved allocation of eye-care resources is another potential benefit of standardized referral guidelines. Glaucoma care is costly; direct glaucoma-related expenditures exceed $3 billion annually in the US alone.^1,13^ Therefore, a systemic approach for delivering care to individuals with manifest glaucoma could aid in the long-term sustainability of glaucoma services, especially as demand for care rises, and the number of ophthalmologists declines.^14,15^

In this study, we aimed to evaluate the outcomes of glaucoma referrals by using data from a large cohort of adults aged 18 to 40 years in the Kaiser Permanente Southern California (KPSC) managed health care system. Kaiser Permanente is the largest integrated managed care consortium in the US; KPSC comprises a network of 13 medical centers serving 4.7 million members as of 2023.^16^ Although KPSC prioritizes preventative care, it lacks standardized evidence-based guidelines for glaucoma referrals. Therefore, there is an opportunity to improve the current referral system, especially for adults aged 18 to 40 years who are generally at low risk of glaucoma compared with adults older than 60 years.^17^ There is also an opportunity to identify systemic deficiencies, such as loss to follow-up of individuals with suspected glaucoma who are at high risk after referral, which could contribute to disparities in glaucoma outcomes.

## Methods

This cohort study was approved by the KPSC Institutional Review Board with a waiver of informed consent because the research was conducted with deidentified data and posed minimal risk to the participants. The study followed the tenets of the Declaration of Helsinki^18^ and complied with HIPAA (Health Insurance Portability and Accountability Act). We followed the Strengthening the Reporting of Observational Studies in Epidemiology (STROBE) reporting guideline.

KPSC members can freely schedule comprehensive eye examinations with an optometrist or receive a referral from a primary care physician to see an ophthalmologist. All KPSC patients undergoing comprehensive eye examinations are evaluated for glaucoma risk factors, such as enlarged CDR or elevated IOP. IOP is checked using a range of contact and noncontact methods and devices, including Goldmann Applanation Tonometry, iCare (Revenio Group), Tono-Pen (Reichert), and Marco Tonoref II M3 (Nidek). Patients with a first-time glaucoma suspect or glaucoma diagnosis are referred for glaucoma evaluation with visual field (VF) and/or optical coherence tomography (OCT) testing. Referrals are made based on clinical suspicion by individual clinicians rather than standardized guidelines issued by KPSC.

Available sociodemographic variables included sex, age at diagnosis, and self-reported race, which was combined with self-reported ethnicity at the time of entry into the EHR. Race and ethnicity data were collected because there are known racial and ethnic differences in glaucoma prevalence and risk. Based on available data, the primary race and ethnicity categories were Asian, Black, Hispanic, White, and other (including American Indian or Alaska Native and Native Hawaiian or Other Pacific Islander). Those who declined to self-report their race and ethnicity were combined under unknown. Available clinical data included glaucoma subtype diagnosis, date of diagnosis, dates of VF and OCT testing, number of visits with eye-care clinicians, IOP, CDR, and refractive error. Disease diagnoses were provided by a wide range of KPSC clinicians using broad definitions rather than standardized study definitions.

### Study Definitions and Data

All KPSC members aged 18 to 40 years who received first-time eye examinations with a KPSC optometrist or KPSC ophthalmologist between January 1, 2013, and December 31, 2018, were identified based on encounter date and internal clinician and specialty codes (eFigure in Supplement 1). Referable glaucoma was defined as a composite diagnosis of glaucoma suspect or any glaucoma subtype based on *International Classification of Diseases, Ninth Revision* (*ICD-9*) and *International Statistical Classification of Diseases and Related Health Problems, Tenth Revision* (*ICD-10*) codes (eTable 1 in Supplement 1) recorded in the Kaiser EHR system (Epic Systems). The index date was defined as the date of the first eye examination for all patients except those with a diagnosis of referable glaucoma that occurred later in the study period. For those patients, the index date was defined as the date of the first diagnosis of referable glaucoma. Inclusion in the study required continuous observability (enrollment with KPSC) of at least 2 years after the index date. Data analysis occurred between September 2022 and August 2024.

The referred group was defined as the subset of patients diagnosed with referable glaucoma by an optometrist or ophthalmologist. The evaluated group was defined as the subset of referred patients who successfully completed VF or OCT testing based on *ICD-9* and *ICD-10* codes within 2 years of the index date (eTable 1 in Supplement 1). The lost-to-follow-up group was defined as the subset of referred patients who did not complete VF or OCT testing within 2 years of the index date. A 2-year window was selected to capture the majority (94.7%) of first VF or OCT testing without excluding patients who may have experienced temporary barriers to care.

The primary outcome measured was diagnosed glaucoma, defined as the presence of any glaucoma subtype diagnosis based on *ICD-9* and *ICD-10* codes (eTable 1 in Supplement 1) at the last visit within 2 years of the index date, occurring on or after the date of VF or OCT testing. In the absence of a glaucoma diagnosis after evaluation, patients remained categorized as those with suspected glaucoma. A secondary outcome measure was loss to follow-up, defined as failing to obtain VF or OCT testing within 2 years of the index date.

CDR, IOP, and refractive error data were derived from the first ophthalmologic or optometric visit in the Kaiser EHR database within the study period. One eye was selected for analysis based on disease severity to avoid mislabeling and analysis of the nonglaucomatous eye due to patient-level diagnosis codes. The eye with a greater CDR or, if a CDR was unavailable, a higher IOP was selected for analysis. If CDR and IOP data were both unavailable, the study eye was chosen at random. The method of tonometry was not considered in this study. Spherical equivalent was calculated as the sum of the sphere power and one-half of the cylinder power and categorized as hyperopia (spherical equivalent > 0.5 diopters [D]), emmetropia (−0.5 < spherical equivalent ≤ 0.5 D), low myopia (−3.0 < spherical equivalent ≤ −0.5 D), and moderate and high myopia (spherical equivalent ≤ −3.0 D).^19^

### Statistical Analysis

The proportion of referred, evaluated, and diagnosed patients within the overall cohort was calculated and stratified by age, sex, and race and ethnicity. Continuous data were expressed as means and SDs and compared using the Wilcoxon rank sum test for 2 groups or Kruskal-Wallis and post hoc Dunn tests for 3 or more groups. Categorical data were expressed as proportions and percentages and were compared using the χ^2^ test. Univariable and multivariable logistic regression analyses were performed for the primary outcome of diagnosed glaucoma (among evaluated patients) and for the secondary outcome of lost to follow-up (among referred patients), with age, sex, race and ethnicity, CDR, IOP, and spherical equivalent as covariates.

A secondary logistic regression analysis was performed by dichotomizing age, IOP, and CDR into high-risk and low-risk categories with diagnosed glaucoma as the outcome measure. Dichotomization was based on means to create groups of relatively equal sizes for easier interpretation and implementation. High-risk age, IOP, and CDR were defined as being at or above the mean, while low-risk age, IOP, and CDR were defined as being below the mean. Four groups were created based on combinations of dichotomized age and IOP (low-risk age and IOP, low-risk age and high-risk IOP, high-risk age and low-risk IOP, and high-risk age and IOP). The probability of detected glaucoma per a 0.1-unit increase in the CDR was plotted for each group, and the negative predictive value and positive predictive value of each group were calculated. The proportion of evaluated patients expected to be under 2.5%, 5.0%, and 10.0% risk of glaucoma based on their age and IOP risk group and CDR was calculated along with the actual proportion in these low-risk categories that met the glaucoma outcome. A manual review of clinic notes from patients diagnosed with glaucoma who were very low risk was conducted to assess the validity of *ICD-9* and *ICD-10* codes. Univariable and logistic regression analyses were conducted with dichotomized age, IOP, and CDR. Clinically significant effects were defined as odds ratio (OR) greater than 1.10 or less than 0.90 and a 1-sided *P* < .05. Statistical analysis was performed using R, version 4.3.1 (R Project for Statistical Computing).

## Results

A total of 292 453 KPSC patients between 18 and 40 years of age met criteria for inclusion (mean [SD] age, 29.8 [6.4] years) (eFigure in Supplement 1). Among 12 050 identified patients with referable glaucoma (4.1%), 52.3% were female, 47.7% were male, 17.7% were Asian, 9.4% were Black, 43.4% were Hispanic, 19.2% were White, and 7.6% were of other race and ethnicity (Table 1). Of these referred patients, 6827 (56.7%) were evaluated with either VF or OCT testing within 2 years. Evaluated patients had greater mean (SD) numbers of ophthalmologic visits (2.2 [4.9]) compared with the general population (0.5 [1.6]) and the referred group (1.7 [4.4]) (*P* < .001). However, they had a similar mean (SD) number of optometry visits (1.5 [1.7]) compared with the general population (1.4 [1.2]; *P* = .44) and the referred group (1.5 [1.6]; *P* = .11). 2344 of 12 050 patients with referable glaucoma (19.5%), 1045 of 6827 patients with evaluated glaucoma (15.3%), and 187 of 563 patients with diagnosed glaucoma (33.2%) had an IOP greater than 21 mm Hg.

**Table 1.** Referred, Evaluated, and Diagnosed Patients With Glaucoma Stratified by Age and Sex

Age range, y	Female				Male				Overall			
	Referred, No.	Evaluated, No.	Diagnosed, No.	With glaucoma, %	Referred, No.	Evaluated, No.	Diagnosed, No.	With glaucoma, %	Referred, No.	Evaluated, No.	Diagnosed, No.	With glaucoma, %
18-19	181	91	2	2.2	196	96	5	5.2	377	187	7	3.7
20-29	2206	1154	48	4.2	1853	974	69	7.1	4059	2128	117	5.5
30-40	3914	2297	173	7.5	3700	2215	266	12.0	7614	4512	439	9.7
Overall	6301	3542	223	6.3	5749	3285	340	10.4	12 050	6827	563	8.2

Among the 6827 evaluated patients, there were 1439 (21.1%) who were Asian, 595 (8.7%) who were Black, 2827 (41.4%) who were Hispanic, 1257 (18.4%) who were White, and 530 (7.8%) who were of other race and ethnicity (eTable 2 in Supplement 1). There were a total of 563 patients (8.2%) who were diagnosed with glaucoma (344 [61.1%] with open angle, 28 [5.0%] with angle closure, 84 [14.9%] with secondary glaucoma, and 107 [19.0%] with unspecified glaucoma). Patients with diagnosed glaucoma had a greater prevalence of moderate and high myopia (40.0% vs 29.0%; *P* < .001), a higher mean (SD) IOP (22.3 [9.8] mm Hg vs 17.2 [4.7] mm Hg; *P* < .001), and a larger mean (SD) CDR (0.7 [0.2] vs 0.6 [0.2]; *P* < .001). On multivariable analysis (Table 2), male sex (OR, 1.55 [95% CI, 1.07-2.27]; *P* = .02), moderate and high myopia (OR, 2.24 [95% CI, 1.29-4.05]; *P* = .005), an elevated IOP (OR, 1.19 [95%CI, 1.15-1.23] per 1 mm Hg; *P* < .001), and a greater CDR (OR, 1.53 [95% CI, 1.34-1.75] per 0.1 unit; *P* < .001) were associated with greater odds of glaucoma (area under the receiver operating characteristic curve, 0.77 [95% CI, 0.73-0.82]; *P* ≤ .02).

**Table 2.** Univariable and Multivariable Logistic Regression Analyses of Baseline Factors Associated With Glaucoma Detection

Characteristic	Total evaluated patients, No.	Evaluated patients diagnosed with glaucoma, No. (%)	Analysis			
			Univariable		Multivariable	
			OR (95% CI)	*P* value	OR (95% CI)	*P* value
Age, y	NA	NA	1.06 (1.04-1.08)	<.001	1.06 (1.03-1.10)	<.001
Sex						
Female	3542	223 (6.3)	1 [Reference]	NA	1 [Reference]	NA
Male	3285	340 (10.4)	1.72 (1.44-2.05)	<.001	1.55 (1.07-2.27)	.02
Race and ethnicity						
Asian	1439	124 (8.6)	1.05 (0.80-1.37)	.75	1.41 (0.73-2.81)	.31
Black	595	87 (14.6)	1.90 (1.40-2.57)	<.001	1.79 (0.88-3.72)	.11
Hispanic	2827	187 (6.6)	0.79 (0.61-1.01)	.06	1.02 (0.56-1.94)	.96
White	1257	104 (8.3)	1 [Reference]	NA	1 [Reference]	NA
Other^a^	530	38 (7.2)	0.86 (0.58-1.25)	.43	0.92 (0.39-2.13)	.86
Unknown	179	23 (12.8)	1.63 (0.99-2.60)	.05	2.04 (0.68-5.45)	.17
Spherical equivalent						
Emmetropia	878	32 (4.1)	1 [Reference]	NA	1 [Reference]	NA
Hyperopia	254	11 (4.3)	1.31 (0.63-2.56)	.44	1.20 (0.43-2.96)	.71
Low myopia	1512	91 (6.0)	1.71 (1.12-2.68)	.02	1.36 (0.78-2.44)	.29
Moderate and high myopia	1041	88 (8.5)	2.45 (1.61-3.85)	<.001	2.24 (1.29-4.05)	.005
IOP, mm Hg	NA	NA	1.12 (1.11-1.14)	<.001	1.19 (1.15-1.23)	<.001
CDR, per 0.1 unit	NA	NA	1.41 (1.30-1.53)	<.001	1.53 (1.34-1.75)	<.001

Abbreviations: CDR, cup-disc ratio; IOP, intraocular pressure; NA, not applicable; OR, odds ratio.

^a^ Includes but is not limited to American Indian or Alaska Native and Native Hawaiian or Other Pacific Islander.

Dichotomization cutoffs were based on the mean (SD) of age (32.0 [6.0] years), IOP (17.6 [5.5] mm Hg), and CDR (0.7 [0.2]). There were 16.0% to 28.4% of the evaluated group among each of the 4 risk groups created using age and IOP. Only 51 of 1613 patients (3.2%) in the low-risk age and IOP group were diagnosed with glaucoma compared with 202 of 1477 (13.7%) in the high-risk age and IOP group. CDR-specific risk of detected glaucoma remained lower than the overall risk (8.2%), with the CDR less than 0.8 for all risk groups except the high-risk age and IOP group (Figure). The multivariable analysis with dichotomized age, IOP, and CDR was similar to the undichotomized model, except Black race remained significantly associated with glaucoma diagnosis (eTable 3 in Supplement 1). Among 309 patients, aged 32 years or older with a CDR of 0.7 or higher and an IOP of 18 mm Hg or higher, there was a 23.3% positive predictive value of glaucoma detection compared with a 2.0% positive predictive value of glaucoma detection among 768 patients in the lowest risk for age, CDR, and IOP (Table 3).

**Figure.**
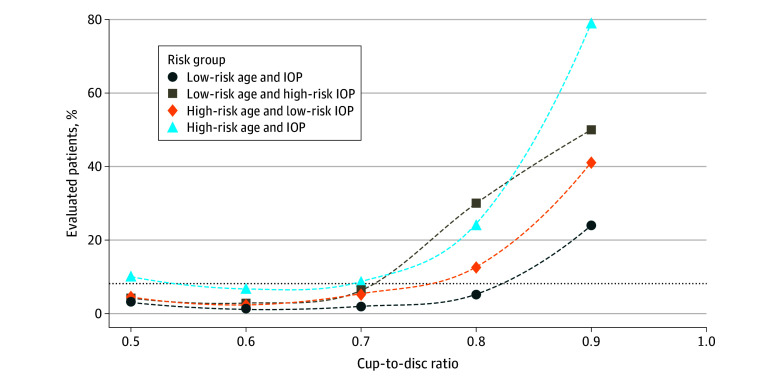
Proportion of Evaluated Patients Detected to Have Glaucoma Within 2 Years After Referable Glaucoma Diagnosis, Stratified by Dichotomized Age and Intraocular Pressure (IOP) Risk Group The overall risk of glaucoma (8.2%) is indicated by the horizontal dotted black line. Low-risk age includes patients younger than 32 years; low-risk IOP, less than 18 mm Hg; high-risk age, 32 years or older; high-risk IOP, 18 mm Hg or more. Patients with a cup-disc ratio less than 0.7 were considered low risk; 0.7 or more was considered high risk.

**Table 3.** Negative and Positive Predictive Values of Age, IOP, and CDR for Glaucoma Detection

Outcome measure	TN or TP, No.	FN or FP, No.	Predictive value, %^a^
**Negative results**			
Age <32 y	2824	182	93.9
IOP <18 mm Hg	3279	185	94.7
CDR <0.7	3049	141	95.6
Age <32 y and IOP <18 mm Hg	1562	51	96.8
Age <32 y and CDR <0.7	1325	38	97.2
IOP <18 mm Hg and CDR <0.7	1813	46	97.5
Age <32 y and IOP <18 mm Hg and CDR <0.7	754	14	98.2
**Positive results**			
Age ≥32 y	391	3440	10.2
IOP ≥18 mm Hg	300	2267	11.7
CDR ≥0.7	192	1505	11.3
Age ≥32 y and IOP ≥18 mm Hg	202	1275	13.7
Age ≥32 y and CDR ≥0.7	128	781	14.1
IOP ≥18 mm Hg and CDR ≥0.7	114	445	20.4
Age ≥32 y and IOP ≥18 mm Hg and CDR ≥0.7	72	237	23.3

Abbreviations: CDR, cup-disc ratio; FN, false negative or number of patients who had an incorrect negative prediction; FP, false positive or number of patients who had an incorrect positive prediction; IOP, intraocular pressure; TN, true negative or number of patients who had a correct negative prediction; TP, true positive or number of patients who had a correct positive prediction.

^a^ The formula for a negative predictive value is TN/(TN + FN); the formula for a positive predictive value is TP/(TP + FP).

Among the 6827 evaluated patients, 28.2% had a predicted risk below 2.5%, 56.4% had a predicted risk below 5.0%, and 91.1% had a predicted risk below 10.0%, based on dichotomous age and IOP and continuous CDR (Figure). Among 325 diagnosed patients with age, IOP, and CDR data, 24 patients (7.4%) had a predicted risk below 2.5%. Upon manual review of clinic notes from the 24 patients with very low risk, 9 (37.5%) received IOP-lowering treatments, 3 (12.5%) were diagnosed with normal tension glaucoma, 3 (12.5%) were diagnosed with secondary or angle-closure glaucoma, 2 (8.3%) were patients with large discrepancies in CDR compared with later visits, and 1 (4.2%) had suspected glaucoma that was miscoded as open-angle glaucoma.

Among the 6827 who received testing within 2 years, 1025 (15.0%) had VF testing only, 1544 (22.6%) had OCT testing only, and 4258 (62.4%) had both VF and OCT. Among 12 050 patients with referable glaucoma, 5223 (43.3%) were lost to follow-up and did not receive VF testing or OCT within 2 years. Although Black race was associated with higher odds (OR, 1.90 [95% CI, 1.40-2.57]; *P* < .001) of being diagnosed with glaucoma compared with White race, on multivariable analysis (Table 4), those with higher odds of being lost to follow-up (area under the receiver operating characteristic curve, 0.68 [95% CI, 0.67-0.70]) included patients who were Black (OR, 1.45 [95% CI, 1.16-1.82]; *P* = .001) and Hispanic (OR, 1.19 [95% CI, 1.01-1.39]; *P* = .04) and those who had a smaller CDR (OR, 1.52 [95% CI, 1.47-1.59] per 0.1 decrease; *P* < .001) and low myopia (OR, 1.15 [95% CI, 1.00-1.33]; *P* = .05).

**Table 4.** Univariable and Multivariable Logistic Regression Analyses of Baseline Factors Associated With Loss to Follow-Up

Characteristic	Total referred patients, No.	Referred patients lost to follow-up, No. (%)	Univariable analysis		Multivariable analysis	
			OR (95% CI)	*P* value	OR (95% CI)	*P* value
Age, y	NA	NA	0.97 (0.97-0.98)	<.001	0.97 (0.96-0.98)	<.001
Sex						
Female	6301	2759 (43.8)	1 [Reference]	NA	1 [Reference]	NA
Male	5749	2464 (42.9)	0.96 (0.89-1.03)	.31	1.11 (0.99-1.25)	.07
Race and ethnicity						
Asian	2130	691 (32.4)	0.57 (0.50-0.64)	<.001	0.83 (0.68-1.02)	.07
Black	1128	533 (47.3)	1.06 (0.92-1.22)	.41	1.45 (1.16-1.82)	.001
Hispanic	5227	2400 (45.9)	1.01 (0.91-1.11)	.91	1.19 (1.01-1.39)	.04
White	2318	1061 (45.8)	1 [Reference]	NA	1 [Reference]	NA
Other^a^	327	148 (45.3)	0.87 (0.75-1.02)	.08	1.05 (0.83-1.35)	.64
Unknown	920	390 (42.4)	0.98 (0.78-1.23)	.86	1.08 (0.75-1.54)	.70
Spherical equivalent						
Emmetropia	1615	737 (45.6)	1 [Reference]	NA	1 [Reference]	NA
Hyperopia	532	278 (52.3)	1.11 (0.91-1.35)	.31	1.18 (0.93-1.49)	.19
Low myopia	3095	1583 (51.1)	1.09 (0.96-1.23)	.19	1.15 (1.00-1.33)	.05
Moderate and high myopia	1863	822 (44.1)	0.82 (0.71-0.93)	.004	0.93 (0.80-1.10)	.45
IOP, mm Hg	NA	NA	1.00 (0.99-1.00)	.33	0.94 (0.93-0.96)	<.001
CDR, per 0.1 unit	NA	NA	0.72 (0.70-0.74)	<.001	0.66 (0.63-0.68)	<.001

Abbreviations: CDR, cup-disc ratio; IOP, intraocular pressure; NA, not applicable; OR, odds ratio.

^a^ Includes American Indian or Alaska Native and Native Hawaiian or Other Pacific Islander.

## Discussion

In this retrospective cohort study of adults aged 18 to 40 years from a large managed health care system, only 8.2% of patients who were referred and received a glaucoma evaluation were diagnosed with glaucoma. Black and Hispanic patients were at higher risk for not receiving glaucoma evaluation after referral. A simple 3-variable model based on age, IOP, and CDR effectively stratified patients with low risk (3.2%) and high risk (13.7%) of being diagnosed with glaucoma. Our model demonstrated that implementing a 2.5% risk threshold for glaucoma referral in adults aged 18 to 40 years could reduce such referrals by 28.2%. This study provided a framework for evaluating the outcomes of glaucoma referrals and establishing standardized risk-stratification guidelines that may improve the equity of eye care and efficiency of resource utilization.

Our findings raise questions about the effectiveness of glaucoma referrals among adults aged 18 to 40 years in the absence of standardized referral guidelines. Previous studies of older adults over 40 years of age, including those in Los Angeles County, reported higher diagnosis rates of up to 25.0% following referral for specialist evaluation.^6,20^ One obvious explanation for the low yield of glaucoma evaluations in our study cohort is the low overall prevalence of glaucoma among these adults aged 18 to 40 years, ranging between 0.16% and 0.4% in previous studies.^21,22^ Additionally, tools developed to risk stratify older adults may not generalize to adults aged 18 to 40 years, leaving clinicians with few options available to discern which patients are at sufficient risk to warrant referral.^3,23^

Given that the KPSC glaucoma-referral process is typically initiated by optometrists, we elected to model only factors that are routinely assessed in general eye-care settings. The lowest-risk group, which satisfied all 3 low-risk criteria (aged <32 years, IOP <18 mm Hg, and CDR <0.7), comprised 11.2% of evaluated patients (n = 768 of 6827) and carried a negative predictive value of 98.2%. These results demonstrate that risk stratification of adults aged 18 to 40 years in the KPSC system is feasible using readily accessible data. Assessing glaucoma risk factors that require specialized equipment, such as visual fields, refractive error, and central corneal thickness, could improve the accuracy of diagnosis but may be time or cost prohibitive in a screening setting.^7,24,25^ Therefore, the marginal diagnostic benefit compared with the cost of assessing each risk factor should be thoroughly considered in the context of available health care resources prior to implementation.

We intentionally avoided ascribing clinical significance to specific glaucoma risk thresholds, as establishing such thresholds is a complex issue that varies by patient population and health care system. Value-based approaches, in which the benefit of a diagnostic test is quantified, using standardized measurements such as quality-adjusted life-year or an incremental cost-effectiveness ratio, have been applied to other complex clinical decisions.^26^ Risk tolerance must also be discussed between the clinician and patient, and decisions to refer should be made on an individualized basis while considering the complete clinical picture. However, implementing evidence-based referral thresholds could have significant resource-conserving benefits. For example, implementing a 5.0% risk threshold could reduce the existing number of KPSC glaucoma referrals among adults aged 18 to 40 years by 56.4%. While KPSC may have sufficient eye-care resources to accommodate a more stringent risk threshold, there are resource-constrained safety-net health care systems that must prioritize the care of a smaller number of individuals with higher glaucoma risk.

Our data suggest that the increased workload of evaluating referable glaucoma falls primarily on ophthalmologists rather than optometrists, a concerning finding given the low yield of evaluations and worsening ophthalmologic workforce shortage.^27^ Patients evaluated for glaucoma had the same number of optometry visits during the study period but a greater number of ophthalmologic visits compared with the adults aged 18 to 40 years in our cohort. This increased use of ophthalmologic appointments may represent a suboptimal allocation of clinician resources, given that 91.8% of all evaluated patients were not diagnosed with glaucoma. Given the significant decline in ophthalmologist density per 100 000 people from 1995 to 2017 in the US, enhancements to glaucoma-referral pathways that enable ophthalmologists to focus on higher-level patient care compared with low-yield evaluations should be considered.^15^ For example, KPSC runs a low-resource teleophthalmologic monitoring program that follows individuals with suspected glaucoma with low risk after initial evaluation and detection.^28^

While there is a need to improve glaucoma-referral practices by decreasing the frequency of evaluations for patients with low risk, some patients with high risk are not receiving evaluations at all. Just under half (43.3%) of the patients in our study (n = 5223 of 12 050) did not return for a glaucoma evaluation after referral, representing a glaring lost opportunity to detect glaucoma. Black and Hispanic patients were at higher risk of being lost to follow-up, even though Black race was associated with higher odds (OR, 1.90 [95% CI, 1.40-2.57]) of being diagnosed with glaucoma compared with White race. These findings are consistent with previous epidemiologic studies and are concerning given disparities in glaucoma detection, and outcomes are widely recognized.^29,30,31,32^ We provide evidence that racial and ethnic disparities may negatively impact the early trajectory of these patients during the critical period preceding their diagnosis, thus delaying potential detection and treatment.^33^ Finally, our findings highlight that health insurance coverage is only 1 of many social determinants of health, including health literacy and cultural beliefs, that may contribute to disparities in eye-care utilization and glaucoma outcomes. Further development of interventions that increase adherence to glaucoma referrals, such as patient education and appointment notifications, is urgently needed.^34^

### Limitations

Our study has several limitations. First, glaucoma diagnoses in our study were derived from *ICD-9* and *ICD-10* codes that may have been affected by misdiagnosis or miscoding errors. This led us to perform an audit of clinic notes from patients diagnosed with glaucoma who were predicted to be at low risk by our model (most likely to be misdiagnosed or miscoded), which revealed only 1 patient with suspected glaucoma that was miscoded as primary open-angle glaucoma. The majority of the remaining patients were previously diagnosed and were receiving IOP-lowering treatment, which is not factored into our models, or experienced normal tension glaucoma, which is rare among adults aged 18 to 40 years.^35^ Overall, these findings are consistent with prior studies supporting the validity of using *ICD* codes to identify patients with glaucoma.^36,37^ Second, *ICD-9* and *ICD-10* codes may not provide accurate information about the laterality of a referable glaucoma diagnosis. Therefore, we opted to include the eye with more severe CDR and/or IOP in our analysis, which may have introduced sampling error. However, 66.1% of the patients in our study with glaucoma were diagnosed with either primary angle-closure glaucoma or primary open-angle glaucoma, which are typically bilateral diseases.^38^ Third, clinical measurements were acquired from free text entries in the EHRs, which sometimes lacked specificity (eg, vertical or horizontal CDR).^4^ This uncertainty is a limitation of current EHR systems and highlights the need for standardized data concepts to represent these measurements.^39^ Fourth, tonometry was performed using a range of different methods and devices in KPSC, which could have contributed to false positives or negatives compared with a single approach.^40^ However, the benefit of standardizing tonometric procedures in the risk-stratification process requires further study prior to widespread implementation. Finally, our window for glaucoma diagnosis spanned only 2 years after the first referable glaucoma diagnosis; therefore, patients with longer intervals before receiving a glaucoma evaluation could have been missed. Similarly, the care of patients whose 2-year windows extended into 2020 could have been disrupted by the COVID-19 pandemic.

## Conclusions

The findings of this cohort study provide insight into the low yield of glaucoma evaluations in an adult population aged 18 to 40 years in the absence of standardized evidence-based referral guidelines. Further work is needed using longitudinal data to assess the optimal frequency of reevaluation for referred and evaluated patients. In addition, efforts to improve glaucoma care must continue to be evaluated for fairness and bias to ensure racial and ethnic minority groups receive equitable care. This study provides a crucial step toward standardizing glaucoma-referral protocols, reducing low-yield eye-care expenditures, and ensuring reproducible and equitable care in diverse patient populations.

## Notes

Supplement 1. **eFigure.** Attrition Diagram and Study Group Definitions **eTable 1.** Referable Glaucoma Diagnosis Codes and Procedural Codes **eTable 2.** Glaucoma Diagnoses at the Last Visit Stratified by Race and Subtype **eTable 3.** Univariable and Multivariable Analysis of Risk Factors for Detection of Glaucoma in Which Predictors Age, Intraocular Pressure (IOP), and Cup-to-Disc Ratio (CDR) Are Dichotomized at the Mean

Supplement 2.
Data Sharing Statement
