# Supplementary material for: Outcomes of Glaucoma Referrals in Adults Aged 18 to 40 Years
Source: JAMA Netw Open. 2025 Feb 6;8(2):e2457843. doi: 10.1001/jamanetworkopen.2024.57843 (PMC11803476; doi:10.1001/jamanetworkopen.2024.57843)
Supplement: Supplement 1. — eFigure. Attrition Diagram and Study Group Definitions eTable 1. Referable Glaucoma Diagnosis Codes and Procedural Codes eTable 2. Glaucoma Diagnoses at the Last Visit Stratified by Race and Subtype eTable 3. Univariable and Multivariable Analysis of Risk Factors for Detection of Glaucoma in Which Predictors Age, Intraocular Pressure (IOP), and Cup-to-Disc Ratio (CDR) Are Dichotomized at the Mean [file jamanetwopen-e2457843-s001.pdf]

Supplemental Online Content

Frediani T, Yoo K, Cho A, et al. Outcomes of glaucoma referrals in adults aged 18 to 40 years. *JAMA Netw Open*. 2025;8(2):e2457843. doi:10.1001/jamanetworkopen.2024.57843

eFigure. Attrition Diagram and Study Group Definitions
eTable 1. Referable Glaucoma Diagnosis Codes and Procedural Codes
eTable 2. Glaucoma Diagnoses at the Last Visit Stratified by Race and Subtype
eTable 3. Univariable and Multivariable Analysis of Risk Factors for Detection of Glaucoma in Which Predictors Age, Intraocular Pressure (IOP), and Cup-to-Disc Ratio (CDR) Are Dichotomized at the Mean

This supplemental material has been provided by the authors to give readers additional information about their work.

© 2025 Frediani T et al. *JAMA Network Open*.

eFigure. Attrition Diagram and Study Group Definitions

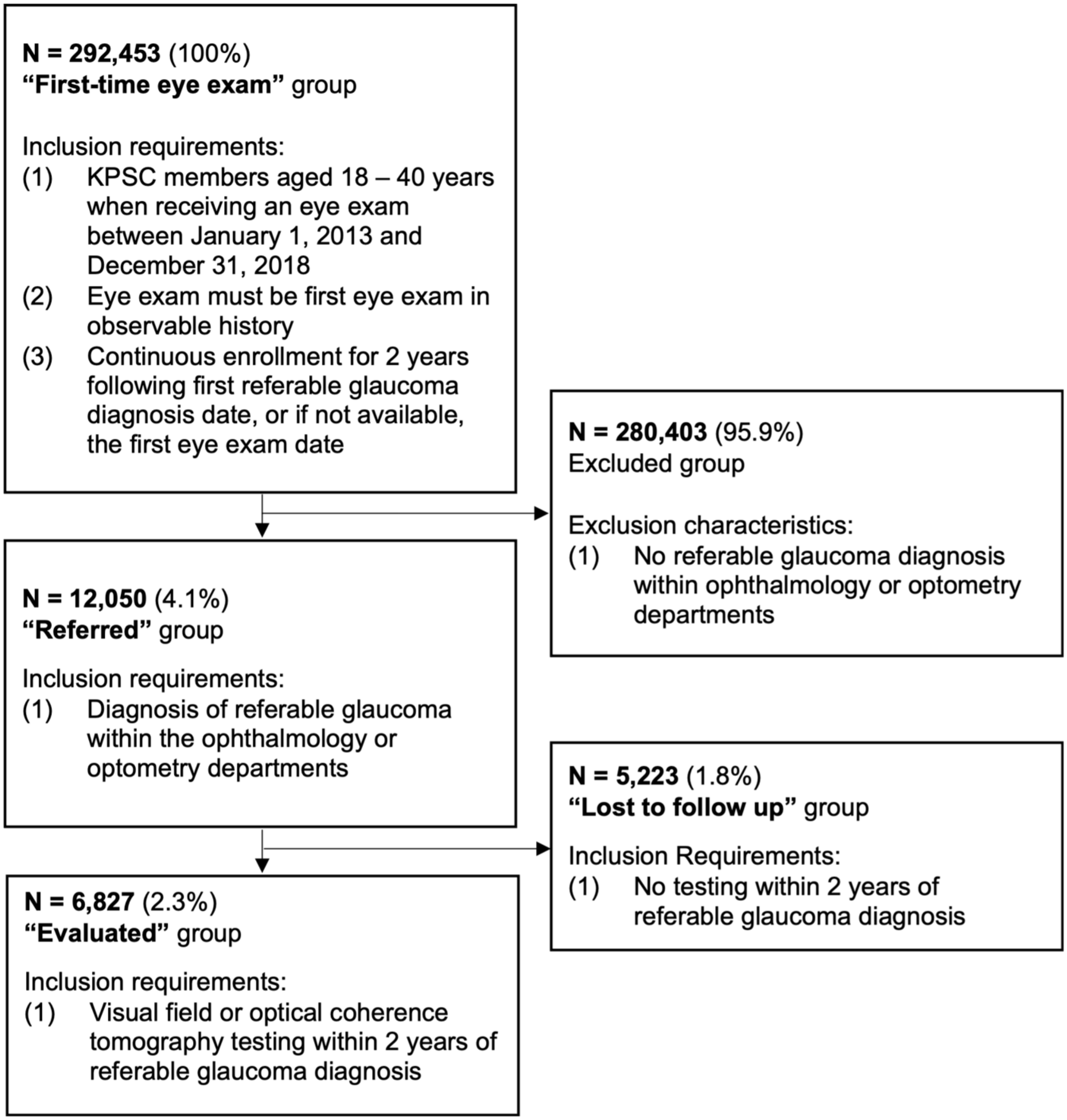

© 2025 Frediani T et al. *JAMA Network Open*.

eTable 1. Referable Glaucoma Diagnosis Codes and Procedural Codes

**Diagnosis Codes**	***ICD9***	***ICD10***
Glaucoma suspect	365.0*<br>377.14<br>365.02<br>365.06	H40.0*<br>H47.23*<br>H40.03*<br>H40.06*
Primary open-angle glaucoma	365.10<br>365.11<br>365.12<br>365.15	H40.10<br>H40.11<br>H40.12<br>H40.15
Primary angle-closure glaucoma	365.2*	H40.2*
Secondary glaucoma	365.13<br>365.3*<br>365.4*<br>365.5*<br>365.6*	H40.13*<br>H40.14*<br>H40.3*<br>H40.4*<br>H40.5*<br>H40.6*
Unspecified glaucoma	365.7*<br>365.8*<br>365.9*	H40.8*<br>H40.9*
**Procedural Codes**	***CPT***	
Visual field testing	92082<br>92083	
Optical coherence tomography	92133	

Abbreviations: ICD9 = International Classification of Diseases, Ninth Revision codes; ICD10 = International Classification of Diseases, Tenth Revision codes; CPT = Current Procedural Terminology.
* Any subsequent code included.

© 2025 Frediani T et al. *JAMA Network Open*.

eTable 2. Glaucoma Diagnoses at the Last Visit Stratified by Race and Subtype

Race	Angle Closure Glaucoma		Open Angle Glaucoma		Secondary Glaucoma		Unspecified Glaucoma	
	N	*%*	*N*	*%*	*N*	*%*	*N*	*%*
Asian	4	14.29%	75	21.80%	21	25.00%	24	22.43%
Black	4	14.29%	64	18.60%	7	8.33%	12	11.21%
Hispanic	14	50.00%	102	29.65%	34	40.48%	37	34.58%
White	4	14.29%	64	18.60%	14	16.67%	22	20.56%
Unknown	0	0.00%	16	4.65%	4	4.76%	3	2.80%
Other*	2	7.14%	23	6.69%	4	4.76%	9	8.41%
**Overall**	28	4.97%	344	61.10%	84	14.92%	107	19.01%

* Participants self-reported their own race, and Other race may include but is not limited to American Indian, Native American, and Native Hawaiian.

© 2025 Frediani T et al. *JAMA Network Open*.

eTable 3. Univariable and Multivariable Analysis of Risk Factors for Detection of Glaucoma in Which Predictors Age, Intraocular Pressure (IOP), and Cup-to-Disc Ratio (CDR) Are Dichotomized at the Mean

				Univariable Analysis		Multivariable Analysis	
		N	n (%)	*OR (CI)*	*P-value*	*OR (CI)*	*P-value*
Age	< 32 years	2996	172 (5.7%)	REF		REF	
	≥ 32 years	3831	391 (10.2%)	**1.87 (1.55-2.25)**	**< 0.001**	**1.73 (1.20-2.50)**	**0.003**
Sex	Female	3542	223 (6.3%)	REF		REF	
	Male	3285	340 (10.4%)	**1.72 (1.44-2.05)**	**< 0.001**	**1.72 (1.20-2.48)**	**0.003**
Race	Asian	1439	124 (8.6%)	1.05 (0.80-1.37)	0.75	1.18 (0.64-2.22)	0.61
	Black	595	87 (14.6%)	**1.90 (1.40-2.57)**	**< 0.001**	**2.01 (1.05-3.90)**	**0.04**
	Hispanic	2827	187 (6.6%)	0.79 (0.61-1.01)	0.06	0.97 (0.56-1.74)	0.90
	White	1257	104 (8.3%)	REF		REF	
	Unknown	179	23 (12.8%)	**1.63 (0.99-2.60)**	**0.05**	1.74 (0.66-4.24)	0.24
	Other	530	38 (7.2%)	0.86 (0.58-1.25)	0.43	0.82 (0.36-1.79)	0.62
SE	Emmetropia	878	32 (4.1%)	REF		REF	
	Hyperopia	254	11 (4.3%)	1.15 (0.54-2.26)	0.71	1.49 (0.56-3.58)	0.40
	Low Myopia	1512	91 (6.0%)	**1.67 (1.11-2.60)**	**0.02**	1.73 (1.01-3.12)	0.06
	Moderate and high myopia	1041	88 (8.5%)	**2.38 (1.57-3.73)**	**< 0.001**	**2.80 (1.62-5.06)**	**< 0.001**
IOP							
	< 18 mmHg	3555	176 (5.0%)	REF		REF	
	≥ 18 mmHg	2570	298 (11.6%)	**2.52 (2.08-3.06)**	**< 0.001**	**3.31 (2.31-4.76)**	**< 0.001**
CDR							
	< 0.7	3192	140 (4.4%)	REF		REF	
	≥ 0.7	1696	192 (11.3%)	**2.78 (2.22-3.49)**	**< 0.001**	**2.97 (2.08-4.25)**	**< 0.001**

Abbreviations: N = Total participants; n = Participants diagnosed with glaucoma; GS = Glaucoma suspect cases; OR = Odds ratio; CI = Confidence interval; SE = Spherical equivalent; IOP = Intraocular pressure; CDR = Cup to disc ratio.
* Participants self-reported their own race, and Other race may include but is not limited to American Indian, Native American, and Native Hawaiian.

© 2025 Frediani T et al. *JAMA Network Open*.
