# Supplementary material for: Outcomes of Glaucoma Referrals in Adults Aged 18 to 40 Years
Source: JAMA Netw Open. 2025 Feb 6;8(2):e2457843. doi: 10.1001/jamanetworkopen.2024.57843 (PMC11803476; doi:10.1001/jamanetworkopen.2024.57843)
Supplement: Supplement 2. — Data Sharing Statement [file jamanetwopen-e2457843-s002.pdf]

Data Sharing Statement

Frediani. Outcomes of Glaucoma Referrals in Adults Aged 18 to 40 Years. *JAMA Netw Open*. Published February 06, 2025. doi:10.1001/jamanetworkopen.2024.57843

Data
Data available: No
